# Family physicians' knowledge and attitudes toward rotavirus vaccination in Muğla, Türkiye

**DOI:** 10.1590/1806-9282.20260176

**Published:** 2026-08-24

**Authors:** Büşra Çakmak, Betul Battaloğlu Inanç

**Affiliations:** 1 Department of Primary Health Care Services, Siirt Provincial Directorate of Health – Siirt, Türkiye.; 2 Muğla Sıtkı Koçman University, Faculty of Medicine, Department of Family Medicine – Muğla, Türkiye.

**Keywords:** Rotavirus infections, Rotavirus vaccines, Family physicians, Health knowledge, attitudes, practice

## Abstract

**OBJECTIVE:**

The aim of this study was to evaluate the knowledge, attitudes, and vaccination practices of family physicians in Muğla, Turkey, regarding rotavirus infection and vaccination.

**METHODS:**

This descriptive cross-sectional study included family medicine residents and family physicians working in primary care settings in Muğla. Data were analyzed using Kruskal-Wallis, Mann-Whitney U, and Pearson chi-square tests. A p<0.05 was considered statistically significant.

**RESULTS:**

Among the participants, 88.6% recommended at least one vaccine not included in the national immunization schedule, with rotavirus vaccine being the most frequently recommended (91.9%). Rotavirus vaccine knowledge scores were significantly associated with age and the recommendation of vaccines not included in the national immunization schedule (p<0.001). Physicians who had vaccinated their own children had significantly higher knowledge scores (p=0.011).

**CONCLUSION:**

Although family physicians demonstrated adequate general knowledge of rotavirus infection, vaccine-related knowledge was insufficient. Knowledge level appears to play a key role in shaping vaccination attitudes and behaviors, highlighting the need for targeted continuing medical education.

## INTRODUCTION

Rotavirus infection is a leading cause of severe acute gastroenteritis in children under 5 years of age worldwide^1,2^. Before widespread vaccination, it was responsible for approximately 440,000 deaths annually. Due to this substantial burden, major organizations such as the World Health Organization and the Centers for Disease Control and Prevention recommend routine rotavirus vaccination for infants^1–4^.

Rotavirus vaccines have been shown to significantly reduce gastroenteritis and related hospitalizations and are included in routine immunization programs in more than 100 countries^1–5^. However, in Turkey, the vaccine is not part of the national immunization schedule.

Vaccination success largely depends on healthcare professionals' knowledge and attitudes. While previous studies have focused on pediatricians, evidence regarding family physicians remains limited^6,7^. Therefore, this study aimed to evaluate the knowledge, attitudes, and practices of family physicians in Muğla regarding rotavirus infection and vaccination.

## METHODS

### Data collection

This descriptive cross-sectional study was conducted between September 16 and October 31, 2024, in the Muğla city center and included family physicians working in Family Health Centers (20 centers comprising 41 family medicine units with 39 active physicians) as well as 38 family medicine residents from the Department of Family Medicine, Muğla Sıtkı Koçman University Faculty of Medicine. The minimum required sample size was determined to be at least 65 participants at a 95% confidence level; a total of 70 individuals participated, while seven declined to participate without providing any reason. Our study was designed to evaluate rotavirus infection and vaccination, which are not included in the national immunization schedule (Diphtheria, Pertussis, Tetanus, Poliomyelitis, Hepatitis B, Hepatitis A, *Haemophilus influenzae* type B, Tuberculosis, Pneumococcal disease, Measles, Mumps, Rubella, and Varicella) and are not administered free of charge. The questionnaire was initially developed based on a literature review and was further refined by incorporating the opinions of specialists and faculty members from the pediatric clinic; it was subsequently reviewed by the Department of Family Medicine, after which the ethical approval process was initiated. A structured questionnaire consisting of 34 items was administered face-to-face; seven items assessed sociodemographic characteristics, while 27 items evaluated knowledge of rotavirus infection and vaccination, as well as vaccination practices.

### Ethical approval

The study was conducted in accordance with ethical principles for scientific research. Ethical approval was obtained from the Muğla Sıtkı Koçman University Faculty of Medicine Ethics Committee (decision date: 14.08.2024, decision number: 240151) and research permission was granted by the Muğla Provincial Health Directorate Scientific Research Review Committee (Reference number: E-15682851-770-254281038). The study was conducted in accordance with the Declaration of Helsinki and in compliance with the ethical standards of our country.

### Statistical analysis

Data were analyzed using SPSS 27. Descriptive statistics were presented as mean, standard deviation, median (QL–QU), frequency, and percentage. Mann-Whitney U, Kruskal-Wallis, Pearson chi-square, and Fisher's exact and Fisher-Freeman-Halton Exact tests were used where appropriate. Spearman correlation was used to assess relationships between variables. A p<0.05 was considered statistically significant.

## RESULTS

In this study of 70 physicians, 37.1% were <30 years, 31.4% were 30–49 years, and 31.4% were ≥50 years; 57.1% were female. Physicians showed good knowledge of core aspects of rotavirus infection, including disease severity (91.7%), symptoms (95.7%), and the absence of specific treatment (95.7%), but had limited knowledge of environmental resistance (44.3%), seasonality (20%), and infectious dose (37.1%). Although awareness of vaccine characteristics was relatively high (82.9–91.4%), notable deficiencies were observed in vaccination schedules (20.0%), contraindications (21.4%), and administration practices (41.4%), as reflected by a high proportion of "uncertain" responses.

Rotavirus vaccine knowledge scores differed significantly by age (Kruskal-Wallis, p=0.016), with higher scores in the 30–49 age group compared to <30 years (Bonferroni-adjusted p=0.012) (Table 1). Significant differences were also found across professional experience groups for both infection and vaccine knowledge (p=0.027 and p=0.014), with ≤3 years of experience associated with lower scores than 4–20 years (adjusted p=0.022 and p=0.011). Duration of family medicine practice was significantly associated with both outcomes (p=0.043 and p=0.004). A further difference was observed between ≤2 and 3–10 years of experience for vaccine knowledge (adjusted p=0.003), but not for infection knowledge (adjusted p=0.057) (Table 1).

**Table 1:** Knowledge scores on rotavirus infection and vaccines according to descriptive characteristics.

Variables		n	Rotavirus infection knowledge score median (QL–QU)	Rotavirus vaccine knowledge score median (QL–QU)
Age group				
	<30 years	26	8 (7–9)	5.5 (4–8)ˣ
	30–49 years	22	9 (8–10)	8 (7–9)
	≥50 years	22	8 (7–9)	7.5 (5–9)
	p^a^		0.073	0.016*
Gender				
	Female	40	8 (7–10)	7 (5–9)
	Male	30	8.5 (7–10)	8 (5–9)
	p^b^		0.814	0.990
Workplace				
	Training and research hospital	35	8 (7–10)	6 (5–9)
	Family health center	35	8 (7–10)	8 (6–9)
	p^b^		0.699	0.233
Professional status				
	Family medicine resident	37	8 (7–10)	7 (5–9)
	Family physician	33	8 (7–10)	8 (6–9)
	p^b^		0.734	0.385
Years of professional experience				
	≤3 years	24	7.5 (7–9)	5.5 (4.5–8.0)
	4–20 years	20	9.5 (8–10)ʸ	9 (6.5–9.0)ʸ
	>20 years	26	8 (7–10)	7 (5–9)
	p^a^		0.027*	0.014*
Duration of family medicine practice				
	≤2 years	30	8 (7–9)	5.5 (5–8)ᶻ
	3–10 years	19	10 (8–10.5)	8 (7.5–9.0)
	>10 years	21	8 (7–9)	8 (4–9)
	p^a^		0.043	0.004*

n: Number of family physicians; QL: first quartile; QU: third quartile;

a Kruskal-Wallis test;

b Mann-Whitney U test;

* p<0.05.

ˣ Post-hoc pairwise comparison (Bonferroni-corrected): Physicians aged <30 years had significantly lower rotavirus vaccine knowledge scores than those aged 30–49 years (adjusted p=0.012).

ʸ Post-hoc pairwise comparison (Bonferroni-corrected): Physicians with ≤3 years of professional experience had significantly lower scores than those with 4–20 years of experience for both rotavirus infection knowledge score (adjusted p=0.022) and rotavirus vaccine knowledge score (adjusted p=0.011).

ᶻ Post-hoc pairwise comparison (Bonferroni-corrected): A significant difference was observed between the ≤2 years and 3–10 years groups for rotavirus vaccine knowledge score (adjusted p=0.003). The corresponding comparison for rotavirus infection knowledge score did not reach statistical significance after Bonferroni correction (adjusted p=0.057).

Among physicians not recommending rotavirus vaccines, 44.5% cited lack of knowledge, 44.5% absence from the national immunization schedule, and 11% cost. Physicians recommending vaccines had significantly higher knowledge scores (p<0.001) (Table 2). Recommending at least one vaccine outside the national schedule was associated with vaccinating one's own children (p=0.018). Vaccination willingness was associated with gender (p=0.010) and practice duration (p=0.013), with lower rates among those with ≥10 years of experience (61.9% vs. 93.3 and 94.7%). No other variables were significant (p>0.05). Physicians' recommendation of the rotavirus vaccine was significantly associated with their intention to vaccinate their own children (Table 3).

**Table 2:** Knowledge scores, demographics, and professional experience according to attitudes toward non-routine vaccines and willingness to vaccinate own children.

Variables		n	Rotavirus infection knowledge score median (QL–QU)	Rotavirus vaccine knowledge score median (QL–QU)
Recommendation of vaccines not included in the national immunization schedule				
	Recommends	62	9 (7–10)	8 (6–9)
	Does not recommend	8	6.5 (4–7)	3.5 (3–4.5)
	p^b^		<0.001	<0.001
Would you vaccinate your own child against rotavirus?				
	Yes	59	9 (7.5–10)	8 (5.5–9)
	Noˣ	8	7 (3.5–7)	3.5 (2.5–6)
	Undecided	3	8 (6–8.5)	8 (6–8.5)
	p^a^		0.002*	0.011*

n: Number of family physicians; QL: first quartile; QU: third quartile.

a Kruskal-Wallis test,

b Mann-Whitney U test.

* p<0.05.

ˣ Post-hoc pairwise comparison (Bonferroni-corrected): Physicians who reported they would not vaccinate their own child had significantly lower scores than those who reported they would, for both rotavirus infection knowledge score (adjusted p=0.002) and rotavirus vaccine knowledge score (adjusted p=0.008).

**Table 3:** Rotavirus vaccination intention for physicians' own children according to their recommendation of rotavirus vaccine.

Recommends rotavirus vaccine	Would not vaccinate own child (No) n (%)	Would vaccinate own child (Yes) n (%)	Undecided n (%)	Total n (%)	p
Does not recommend (n=13)	4 (30.8%)^a^	8 (61.5%)^b^	1 (7.7%)^ab^	13 (18.6%)	0.029*
Recommends (n=57)	4 (7.0%)^a^	51 (89.5%)^b^	2 (3.5%)^ab^	57 (81.4%)	
Total (n=70)	8 (11.4%)	59 (84.3%)	3 (4.3%)	70 (100%)	

n: Number of family physicians; %: Row percentage within each recommendation group.

* p<0.05; Fisher-Freeman-Halton Exact Test.

Superscript letters (a, b) denote subsets of "Would you vaccinate your own child?" categories whose column proportions do not differ significantly from each other at the 0.05 level (Bonferroni-adjusted pairwise comparisons).

## DISCUSSION

It was determined that family physicians had sufficient knowledge regarding the general clinical features and disease burden of rotavirus infection; however, significant knowledge gaps were identified in more detailed epidemiological and clinical aspects. Although they were aware of the general characteristics of vaccines, important deficiencies were detected in vaccination schedules, contraindications, and practical applications. These findings were found to be consistent with studies conducted in Türkiye^6,7^, and other countries^1,2,5,8^. Although rotavirus is known to be an important cause of severe and potentially fatal gastroenteritis in children under five years of age, its absence from the national immunization schedule, issues related to implementation and accessibility, low vaccine acceptance among physicians, specific knowledge gaps regarding the vaccine and the infection, uncertainties about the benefits, risks, and cost-effectiveness of the vaccine, as well as inadequate practices regarding informing parents and including parental participation during vaccination, are among the factors contributing to negative attitudes^1,2,5–7,9,10–14^. Despite the World Health Organization recommending the inclusion of the rotavirus vaccine in routine childhood immunization programs, it has not yet been incorporated into the national immunization schedule in Türkiye^6,7^. Two vaccines are licensed for use in Türkiye. Knowledge gaps were observed regarding appropriate timing and recommended age limits^2–4,10^, administration methods, contraindications, and how to continue the vaccination series when the same vaccine brand is unavailable^6,12^. On the other hand, awareness that rotavirus vaccines are administered orally^11^ indicates that physicians are familiar with some practical aspects of vaccine administration. However, despite the recommendation that the dose should not be repeated if the infant vomits or regurgitates after vaccination, incorrect responses to this item^6,7,12^ indicate ongoing knowledge deficiencies regarding the rotavirus vaccine. These deficiencies may be related to the fact that the vaccine is not included in the Ministry of Health's routine immunization schedule and that physicians are not obligated to administer it. Indeed, studies conducted in Türkiye have reported variability in the rates of recommending the rotavirus vaccine^15^; similar trends have also been observed in the United States^10^, Canada^11^, and Italy^1,2^, this suggests that knowledge and awareness are key determinants in vaccine recommendation and implementation.

Other studies conducted in Türkiye have shown that the rotavirus vaccine is the most frequently recommended optional vaccine by physicians, followed by influenza, meningococcal, HPV, and adult pertussis vaccines^12,16,17^. In our study, however, the order of recommendation was rotavirus, meningococcal, HPV, influenza, and adult pertussis vaccines. This finding suggests that when recommending optional vaccines, physicians may consider not only their level of knowledge but also perceived disease severity and clinical prioritization. In our study, it was also considered that physicians who reported not recommending any vaccines largely adhered to existing guidelines and programs, may have insufficient awareness, knowledge, or confidence regarding vaccines outside the routine schedule^15,16,18,19^, or may perceive the cost, which is not publicly funded, as a barrier to vaccine recommendation^15,17,19,20^. Although cost-effectiveness studies have demonstrated that rotavirus vaccination reduces hospitalizations^5,6,13^, the limited number of such studies in Türkiye may lead physicians to hesitate in recommending the vaccine due to cost-related concerns; however, failure to recommend the vaccine may also result in increased disease burden and hospital costs^20^.

In this context, the level of knowledge and awareness affects vaccine recommendation behavior, and in our study, it was shown that female physicians were more likely to recommend the vaccine, and that physicians who recommended vaccines had significantly higher rates of vaccinating their own children and intention to do so^8^. This finding indicates consistency between physicians' professional recommendations and personal health behaviors, and that the level of knowledge and trust in the vaccine is reflected in both clinical practice and individual decisions^8–14^, as well as that physicians' personal vaccination attitudes influence their professional recommendations^16,21–23^.

In contrast, the higher rate of not vaccinating their own children among physicians who do not recommend the vaccine suggests that factors such as lack of knowledge or the absence of the vaccine from the national immunization schedule^8–12,21,23^, duration of family medicine practice^12,21–23^, low confidence in new vaccines, limited exposure to continuing medical education on these vaccines, or encountering fewer severe rotavirus cases early in their careers may also be associated with lower vaccination rates^17,20–23^. Therefore, increasing educational opportunities for senior physicians who may have become distanced from clinical practice, as well as vaccination programs, management of incomplete vaccination schedules, and effective communication with parents regarding cost and disease burden, play a critical role in this process^16,21^.

Although the sample size is limited, the study represents a substantial proportion of family physicians actively working in primary care and the affiliated training and research hospital, reflecting real-life clinical practice and contributing to the limited literature. However, small subgroup sizes (n=8) reduce statistical power and increase the risk of Type I and II errors. Therefore, the findings should be interpreted with caution and confirmed by larger, prospective, multicenter studies.

## Data Availability

The datasets generated and/or analyzed during the current study are available from the corresponding author upon reasonable request.
